# Trajectories of Emotional Symptoms and Peer Relationship Problems in Children after Nuclear Disaster: Evidence from the Fukushima Health Management Survey

**DOI:** 10.3390/ijerph15010082

**Published:** 2018-01-06

**Authors:** Misari Oe, Masaharu Maeda, Tetsuya Ohira, Shuntaro Itagaki, Mayumi Harigane, Yuriko Suzuki, Hirooki Yabe, Seiji Yasumura, Kenji Kamiya, Hitoshi Ohto

**Affiliations:** 1 Department of Neuropsychiatry, School of Medicine, Kurume University, Fukuoka 830-0011, Japan; 2 Radiation Medical Science Center for the Fukushima Health Management Survey, Fukushima 960-1295, Japan; masagen@fmu.ac.jp (M.M.); teoohira@fmu.ac.jp (T.O.); harigane@fmu.ac.jp (M.H.); yasumura@fmu.ac.jp (S.Y.); kkamiya@fmu.ac.jp (K.K.); hit-ohto@fmu.ac.jp (H.O.); 3 Department of Disaster Psychiatry, School of Medicine, Fukushima Medical University, Fukushima 960-1295, Japan; 4 Department of Epidemiology, School of Medicine, Fukushima Medical University, Fukushima 960-1295, Japan; 5 Department of Neuropsychiatry, School of Medicine, Fukushima Medical University, Fukushima 960-1295, Japan; itasyun@fmu.ac.jp (S.I.); hyabe@fmu.ac.jp (H.Y.); 6 Department of Adult Mental Health, National Institute of Mental Health, National Center of Neurology and Psychiatry, Kodaira 187-8553, Japan; yrsuzuki@ncnp.go.jp; 7 Department of Public Health, School of Medicine, Fukushima Medical University, Fukushima 960-1295, Japan

**Keywords:** Anxiety, Depression, Physical Exercise, Child & Adolescent Psychiatry

## Abstract

The Fukushima Daiichi Nuclear Power Plant accident, which occurred in March 2011, is having long-term effects on children. We planned this study to describe the trajectories of emotional symptoms and peer relationship problems in children and to examine potential risks and protective factors over the 35 months following the accident. The sample was 11,791 children in the first to sixth elementary grades. We identified four patterns for emotional symptoms and three patterns for peer relationship problems, using group-based trajectory modelling. For emotional symptoms, female gender, experience of tsunami and nuclear plant accident, out-of-prefecture evacuees, and insufficient physical activity were associated with the very severe trajectory. In contrast, for peer relationship problems, male gender, experience of nuclear plant accident, and insufficient physical activity were associated with the very severe trajectory. Different factors might be related to the very severe trajectories of emotional symptoms and peer relationship problems.

## 1. Introduction

The Great East Japan Earthquake (GEJE) and Tsunami, which occurred in 2011, is having longitudinal effects on the lives of residents, including children. One study showed that the traumatic symptoms of children improved 20 months after the disaster [1], while another reported that one in four children still had behavior problems, including internalizing and externalizing problems, even two years after the GEJE [2]. A third study revealed that the rates of children with difficulties remained high, even 30 months after the disaster [3]. A more recent study showed that approximately one in three young children in the affected communities exhibited post-traumatic stress disorder (PTSD) symptoms two years after the GEJE [4]. In the Fukushima prefecture, the situation has been more complex because the GEJE and Tsunami caused the Fukushima Daiichi Nuclear Power Plant accident. A recent systematic review showed that mental health problems seemed to be more severe for residents of Fukushima than for those in other affected areas [5]. Mental health status of children in the Fukushima prefecture has been evaluated through the Fukushima Health Management Survey [6], which is held annually. In a cross-sectional study, 22.0% of children of primary school age (6–12 years old) were at risk (the total difficulty score of the Strength and Difficulties Questionnaire ≥ 16) in the fiscal year 2011, and 15.8% were at risk in the fiscal year 2012 [7]. However, longitudinal trajectories of the mental health consequences after the disaster of children in Fukushima have not been examined. 

This study was aimed to describe longitudinal trajectories for emotional symptoms and peer relationship problems after the disaster, and to examine potential risks and protective factors of a severe trajectory. We hypothesized that children’s physical activity is a protective factor for the severe trajectory of emotional symptoms and peer relationship problems after the Fukushima disaster, because the literature concerning the association between physical activity and mental health in adolescence has grown recently [8,9,10].

## 2. Materials and Methods 

This study was designed as a cohort study at three time points.

### 2.1. Study Population

The target population was 11,791 children born after 2 April 1998 and before 1 April 2004 who were elementary school students (i.e., in the first to the sixth grade) on 11 March 2011, and living in one of the 13 municipalities that were the target area of the Mental Health and Lifestyle Survey. The Mental Health and Lifestyle Survey is one of the detailed surveys included in the Fukushima Mental Health Management Survey [6]. The target zone included Hirono, Naraha, Tomioka, Kawauchi, Okuma, Futaba, Namie, Katsurao, Iitate, Minamisoma, Tamura, Kawamata, and hot-spot (places associated with high levels of radiation) areas in Date. The assessments in 2011 and 2012 of the fiscal year, which begins on 1 April and ends on 31 March, were each held in January of the subsequent year; the fiscal year assessment for 2013 was held in February 2014. These assessments were conducted by mail, 10, 22, and 35 months after the disaster. Parents of the children completed the questionnaire. All study participants (i.e., parents of the target children) gave their informed consent for inclusion before they participated in the study. The study was conducted in accordance with the Declaration of Helsinki, and the protocol was approved by the Ethics Review Committee of Fukushima Medical University (No. 1316) and by the Ethical Committee of Kurume University (No. 15188). Reminders were sent once to the parents for each assessment. Response rates were 63.6% in 2011, 39.0% in 2012, and 32.4% in 2013. The number of individuals who responded at least once to any of the three assessments was 8282, or 70.2% of the target population.

### 2.2. Assessments

Experiences of the disaster in the target area were categorized into earthquake, tsunami, and nuclear power plant accident. The experience of nuclear power plant accident was registered if the parents had heard the sound of the nuclear plant explosion.

Emotional symptoms and peer relationship problems were assessed with 10 items of the Strength and Difficulties Questionnaire (SDQ) [11,12,13]. SDQ is a 25-item questionnaire used for identifying psychopathological problems in children. It comprises five subscales: emotional symptoms, conduct problems, hyperactivity/inattention, peer relationship problems, and prosocial behavior. Each item is scored 0, 1, or 2; each subscale score ranges from 0 to 10. The Japanese version of the SDQ has shown adequate internal consistency (α = 0.81) [14,15] and convergent validity [15]. The emotional symptoms subscale and the peer relationship problems subscale were used for this study. 

Exercise habits were evaluated with the question, “How frequent does your child usually get exercise, excluding exercises in physical education class for elementary and middle school children?” There were four response options: almost every day, two to four times a week, once a week, and seldom or never. Questions about sociodemographic characteristics and disaster-related variables were also included.

### 2.3. Analysis Plan

To find heterogeneity in the longitudinal patterns of emotional symptoms and peer relationship problems, we conducted semi-parametric group-based modelling, using SAS software, V.9.4 (SAS, Cary, NC, USA) with the user-written procedure PROC TRAJ [16,17]. The Bayesian information criterion (BIC) and Akaike’s information criterion (AIC) were used to select the best-fitting model. For criteria of trajectory membership, we chose 5% membership because our aim in this study was to understand the whole picture of the trajectories. Each trajectory was labelled by symptom severities, with reference to the Japanese population. (Data are available from “Information for researchers and professionals about the Strengths & Difficulties Questionnaires” website, http://www.sdqinfo.org/). We conducted logistic regression analyses to examine the risk factors of the very severe trajectory group. In all analyses, a 2-tailed *p*-value less than 0.05 was considered significant. Available case analysis was conducted for missing data.

## 3. Results

### 3.1. Sociodemographic Characteristics and Disaster-Related Variables

Sociodemographic characteristics and disaster-related variables are shown in Table 1. Among the children, 11.7% had experienced the tsunami, and 39.7% had heard the sound of the nuclear plant explosion. At the time of first assessment in 2011, 20.3% of the children were out-of-prefecture evacuees.

### 3.2. Trajectories of the Emotional Symptoms and the Peer Relationship Problems

**Figure 1:**
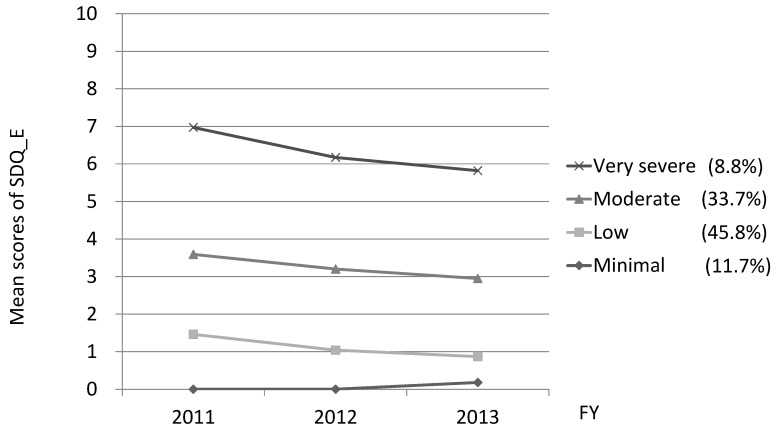
Trajectories of the four-group model of emotional symptoms. The mean scores of emotional symptoms of the four-group (very severe, moderate, low, minimal), using semi-parametric group-based modelling.

The mean scores for emotional symptoms were 2.48 (SD 2.38) in 2011, 2.08 (SD 2.20) in 2012, and 1.92 (SD 2.11) in 2013. Independent sample *t*-tests revealed that the scores for girls were significantly higher than those for boys throughout all assessments (see Table S1). Comparing goodness-of-fit for models with different numbers of trajectories of emotional symptoms over time, a four-trajectory model was found to have the best fit (AIC, −30476.99; BIC, −30533.12). The four trajectories (very severe, moderate, low, minimal) are shown in Figure 1. More than half of respondents belonged to the minimal- or low-symptom trajectory groups, which scored under two points in all three assessments. Decline patterns were observed in all trajectory groups except the minimal-symptom group. A total of 8.8% of the respondents were categorized into the very severe trajectory group, whose average scores were 7.0 in 2011, 6.2 in 2012, and 5.8 in 2013.

**Figure 2:**
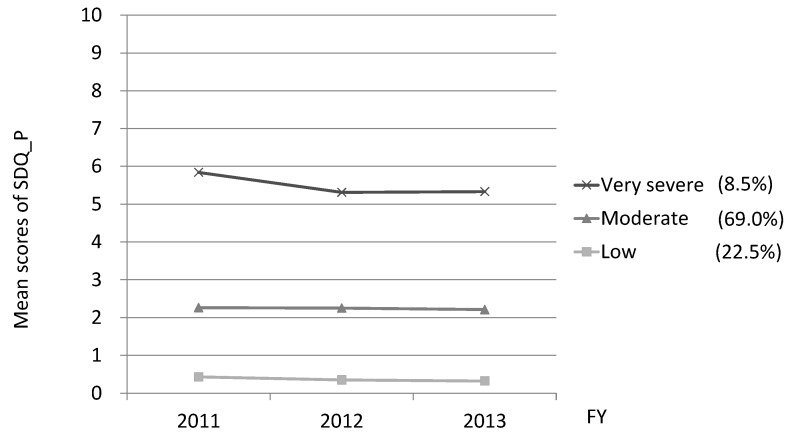
Trajectories of the three-group model of peer relationship problems. The mean scores of peer relationship problems of the three-group (very severe, moderate, low), using semi-parametric group-based modelling.

The mean scores for peer relationship problems were 2.13 (SD1.83) in 2011, 2.06 (SD1.77) in 2012, and 2.07 (SD1.81) in 2013. Independent samples *t*-tests revealed that the score of boys was significantly higher than those of girls in 2012, *t* (7403) = 2.99, *p* < 0.01, however, no significant gender differences in 2011 and 2013. Comparing goodness-of-fit for models with different numbers of trajectories of peer relationship problems over time, a three-trajectory model displayed the best fit (AIC, −29,487.45; BIC −29,529.54). The three trajectories (very severe, moderate, and low) are shown in Figure 2. Around 70% of the respondents were categorized into the moderate trajectory group, with average scores over two points. A total of 8.5% of the respondents belonged to the severe trajectory group, whose average scores were 5.8 in 2011, 5.3 in 2012, and 5.3 in 2013.

### 3.3. Sociodemographic Characteristics by Trajectory Group

Sociodemographic characteristics for each trajectory group are shown in Table S2. Although there are significant differences between sociodemographic characteristics and the trajectory groups for emotional symptoms, only gender, experience of the nuclear plant accident, and exercise habits in 2011 were significantly different among the trajectory groups for peer relationship problems.

### 3.4. Factors Related to the ‘Very Severe’ Trajectory Group

To explore the factors related to the very severe trajectory group, both for emotional symptoms and peer relationship problems, we conducted logistic regression analysis using a forced entry method. Variables considered in the model were experience of disaster (tsunami, nuclear plant accident), living place in 2011 (in or out of Fukushima prefecture), and exercise habits in 2013 (‘very little’ or ‘once a week or more’). We adjusted for gender and age as potential confounders (Table 2). For emotional symptoms, female gender, experience of tsunami and nuclear plant accident, out-of-prefecture evacuation, and a habit of ‘very little’ exercise showed significant effects. For peer relationship problems, male gender, experience of nuclear plant accident, and a habit of ‘very little’ exercise were significant factors.

## 4. Discussion

We identified four trajectories of emotional symptoms, most of which showed gradual improvement. We also found that experience of the disaster and area of residence were associated with the severe trajectory for emotional symptoms. For comparison, a study six months after a wildfire disaster reported that 22.6% of the children studied scored in the abnormal range on emotional symptoms of the SDQ, which reflected the emotional sequelae of the natural disaster [18]. Previous studies have also shown a weak correlation between PTSD symptoms and SDQ [18,19]. Because we lacked information on the children’s PTSD symptoms, we could not describe the symptom structure between PTSD and the emotional symptoms. 

For peer relationship problems, the three trajectories were identified. Despite the very severe trajectory group showed a slight decrease between the 2011 and 2012 assessment, the moderate and low trajectory group seemed no changes among three assessments. One of the most important findings of this study was that the mean scores for peer relationship problems were higher than those in the previously published population studies (see below). For example, in normative data among Japanese school-aged children [15], the mean scores for peer problems by parent ratings were 1.44–1.52, and scores above four were observed only in 4.8% (7–9 yrs) and 5.5% (10–12 yrs) of the Japanese population sample. (Data are available from the “Information for researchers and professionals about the Strengths & Difficulties Questionnaires” website, http://www.sdqinfo.org/). Meanwhile, the mean score for peer relationship problems in a study with children in the tsunami-affected area 20 months after the GEJE [3] was almost same of the mean score in our study. These results suggest that peer relationship problems may be having severe and long-lasting psychological effects on our target children. A 2015 study by Caci et al. [20] demonstrated that the Peer Problems-Specific factor of SDQ mainly reflects a preference for solitude, which could be associated with adjustment difficulties in early adolescence [21].

We found gender differences, both for emotional symptoms and peer relationship problems. These differences, which were dominant in females for emotional symptoms and dominant in males for peer relationship problems, are in line with the normative data among school-aged children in Japan [15], and in European countries [22,23]. These tendencies seem to be amplified in our results in multivariable logistic regression analysis of the severe trajectory group. There are few studies which assessed gender differences among primary school children after psychological trauma. A study on peer victimization in primary schools showed that boys are more likely to be peer victims than girls; however, relational victimization was most strongly associated with PTSD symptoms among girls, but not among boys [24]. Another study conducted in Kuwait revealed that boys are likely to be physical and relational victims [25]. By contrast, it has been known that girls are more at risk for emotional difficulties [26].

The association between exercise habits and the very severe trajectories, both for emotional symptoms and peer relationship problems, was also found in this study, as we hypothesized. Our results were consistent with previous studies showing that more physical activity is related to better mental health [8,9,10]. A four-year (ages 11–15 years) prospective study demonstrated that physical activity was inversely related to mental health problems, especially in boys [8]. Another longitudinal study of adolescents showed that the number of hours spent in physical activity per week at ages 15–16 was negatively associated with emotional symptoms and peer problems in boys at ages 18–19 [9]. A negative association between physical activity and social problems was also reported [27,28,29]. 

Our results revealed that out-of-prefecture evacuation is a risk factor for the very severe trajectory for emotional symptoms of the target children. A study of adult residents after the disaster in Fukushima has shown related results, of higher levels of psychological distress and post-traumatic stress among residents whose address at that time was outside of Fukushima prefecture compared with those who were living in Fukushima prefecture [30]. Another study of residents who had evacuated to Saitama Prefecture or Tokyo Metropolis from Fukushima after the GEJE compared factors related to psychological stress at one year and two years after the accident. The findings showed that the causes of stressors changed during this time, from the damage caused by the earthquake disaster itself to the circumstances of shelter life over time, such as difficult economic conditions, aggravated health conditions, negative labeling as an evacuee, and dissatisfaction with relationships with family and neighbors [31].

In addition, although we do not have data about the mental health symptoms of the parents of the target children, the concerns of parents or parent adverse mental health status might reflect the internalizing symptoms of their children. One study conducted 11 years after the Chernobyl accident showed that the evacuee mothers of young children rated their children’s well-being as significantly worse, despite no statistically significant differences in a teachers’ rating scale [32]. A study by Japanese researchers after the Niigata-Chuetsu earthquake also demonstrated that adverse parental mental health status was associated with PTSD-related behavioral changes in children [33]. It is also likely that the proportion of time spent in solitude or the level of stigma might be higher for the out-of-prefecture evacuees; this could result in difficulty in maintaining peer relationships. 

Our finding that peer relationship problems in children were severe and long-lasting suggests a possible need for healthcare providers to implement an intervention focused on the improvement of peer relationships, both for the children and the parents. Such an intervention could include psychoeducation on the importance of physical activity for physical and psychological development. Making this information available online could also be helpful for the out-of-prefecture evacuees.

Several study limitations require consideration. First, parent-completed questionnaires may be less accurate compared with clinician-administered diagnostic tools. Second, among the SDQ subscales, the peer problems scale has demonstrated lower internal consistency values for both parent and teacher ratings than other subscales [34]. Third, the age range of the target children is wide (6–12 years). It is likely that the children in middle childhood and those in early adolescence have different characteristics. Fourth, we did not have a comparison group which was recruited from not-disaster-affected areas. Fifth, owing to the relatively low response rates, one should not overgeneralize the results. Sixth, we did not have pre-disaster data of the target children. Seventh, some predictors which were indicated as covariates, such as age and gender, may be potential cofounders.

Despite these limitations, our study has several implications for future research and intervention. Different factors were related to the very severe trajectories of emotional symptoms and peer relationship problems. It may be important to facilitate programs with physical activities. Behavioral activation is a potentially helpful candidate intervention, as it has demonstrated significant positive effects on mental and physical health complaints associated with radiation stress among mothers with preschool children in Fukushima [35].

## 5. Conclusions

In conclusion, this study demonstrated the characteristics associated with various trajectories of emotional symptoms and peer relationship problems after the Fukushima nuclear disaster. Future research, including the continuation of the mental health and lifestyle survey as a part of the Fukushima Health Management Survey, is much needed.

## Figures and Tables

**Table 1:** Sociodemographic characteristics of the study sample (*n* = 8282).

Gender	
Male	4213
Female	4069
Age at time of disaster (yrs)	
6	79
7	1340
8	1362
9	1420
10	1395
11	1390
12	1296
Experience of disaster	
Earthquake	7422
Tsunami	879
Heard the sound of the nuclear plant explosion	2971
Missing	797
Living place (current address) in 2011	
In Fukushima prefecture	5937
Out of Fukushima prefecture	1513
Missing	832
Exercise habits in 2011	
Almost every day	939
2–4 times a week	1500
Once a week	1081
Very little	3967
Missing	795

**Table 2:** Multivariate logistic regression analysis of the very severe trajectory group, for emotional symptoms and for peer relationship problems.

Predictor	OR (95% CI) for Emotional Symptoms	OR (95% CI) for Peer Relationship Problems
Gender		
Female	1.22 (1.03–1.45) *	0.66 (0.55–0.78) **
Age at time of disaster		
≤9 years	1.04 (0.88–1.24)	0.90 (0.76–1.07)
Experience of disaster		
Tsunami	1.37 (1.08–1.73) *	1.16 (0.91–1.48)
Heard the sound of the nuclear plant explosion	1.69 (1.42–2.01) **	1.21 (1.02–1.44) *
Living place (current address) in 2011		
Out of Fukushima prefecture	1.25 (1.02–1.52) *	1.05 (0.85–1.28)
Exercise habits in 2011		
Very little	1.48 (1.24–1.77) **	1.60 (1.34–1.90) **

* *p* < 0.05, ** *p* < 0.01.

## Supplementary Materials

The following are available online at www.mdpi.com/1661-7827/15/1/82/s1, Table S1: Gender differences in emotional symptoms, Table S2: Sociodemographic characteristics among the trajectory groups.
