# Supplementary material for: Trajectories of Emotional Symptoms and Peer Relationship Problems in Children after Nuclear Disaster: Evidence from the Fukushima Health Management Survey
Source: Int J Environ Res Public Health. 2018 Jan 6;15(1):82. doi: 10.3390/ijerph15010082 (PMC5800181; doi:10.3390/ijerph15010082)
Supplement: Supplementary file 1 [file ijerph-15-00082-s001.pdf]

Table S1. Gender differences in emotional symptoms

	Girls			Boys				
Fiscal Year	N	Mean	SD	N	Mean	SD	*t*-value	P
2011	3633	2.58	2.42	3798	2.40	2.35	-3.20	0.001
2012	2239	2.22	2.25	2320	1.94	2.14	-4.39	0.000
2013	1872	2.06	2.16	1899	1.78	2.05	-3.99	0.000

Table S2 Sociodemographic characteristics among the trajectory groups

a) The emotional symptoms

**Groups**	**Minimum**	**Low**	**Moderate**	**Very severe**			
	**N(%)**	**N(%)**	**N(%)**	**N(%)**	df	χ^2	p
**Gender**							
Female	294 (41.5)	2005 (48.0)	1420 (51.5)	350 (54.9)	3	33.4	<0.001
Male	415 (58.5)	2173 (52.0)	1337 (48.5)	288 (45.1)			
**Age at the time of disaster**							
9 years or younger	328 (46.3)	2033 (48.7)	1503 (54.5)	337 (52.8)	3	29.8	<0.001
10 years or older	381 (53.7)	2145 (51.3)	1254 (45.5)	301 (47.2)			
**Experience of disaster**							
Tsunami (+)	71 (10.6)	401 (10.6)	312 (12.6)	95 (16.3)	3	18.9	<0.001

Tsunami (-)	598 (89.4)	3365 (89.4)	2156 (87.4)	487 (83.7)			
Heard the sound of nuclear plant explosion (+)	242 (36.2)	1399 (37.1)	1023 (41.5)	307 (52.7)	3	58.3	<0.001
Heard the sound of nuclear plant explosion (-)	427 (63.8)	2367 (62.9)	1445 (58.5)	275 (47.3)			
**Living place in 2011**							
Outside Fukushima prefecture	116 (17.5)	705 (18.8)	545 (22.2)	147 (25.2)	3	22.3	<0.001
Inside Fukushima prefecture	547 (82.5)	3042 (81.2)	1911 (77.8)	437 (74.8)			
**Exercise habit in 2011**							
Very little	293 (43.9)	1883 (50.0)	1424 (57.7)	367 (62.7)	3	80.1	<0.001
Once a week or more	375 (56.1)	1883 (50.0)	1044 (42.3)	218 (37.3)			

b) The peer relationship problems

**Groups**	**Low** **N(%)**	**Moderate** **N(%)**	**Very severe** **N(%)**	df	χ^2	p
**Gender**						
Female	911 (48.9)	2872 (50.2)	286 (40.8)	2	22.3	p<0.001
Male	952 (51.1)	2846 (49.8)	415 (59.2)			

**Age at the time of disaster**						
9 years or younger	944 (50.7)	2913 (50.9)	344 (49.1)	2	0.9	0.65
10 years or older	919 (49.3)	2805 (49.1)	357 (50.9)			
**Experience of disaster**						
Tsunami (+)	183 (10.6)	612 (11.9)	84 (13.7)	2	4.5	0.11
Tsunami (-)	1538 (89.4)	4541 (88.1)	527 (86.3)			
Heard the sound of nuclear plant explosion (+)	626 (36.4)	2073 (40.2)	272 (44.5)	2	14.5	0.001
Heard the sound of nuclear plant explosion (-)	1095 (63.6)	3080 (59.8)	339 (55.5)			
**Living place in 2011**						
Outside Fukushima prefecture	356 (20.8)	1026 (20.0)	131(21.5)	2	1.1	0.6
Inside Fukushima prefecture	1355 (79.2)	4105 (80.0)	477 (78.5)			
**Exercise habit in 2011**						
Very little	760 (44.2)	2825 (54.8)	382 (62.4)	2	81.7	<0.001
Once a week or more	959 (55.8)	2331(45.2)	230 (37.6)			
